# Dataset on Online mass media engagements on YouTube for terrorism related discussions

**DOI:** 10.1016/j.dib.2018.12.020

**Published:** 2019-01-18

**Authors:** Tolulope Kayode-Adedeji, Olusola Oyero, Stella Aririguzoh

**Affiliations:** Department of Mass Communication, Covenant University, Ota Ogun State, Nigeria

## Abstract

The data examined 150 mass media YouTube videos on Al-Shahab, Boko Haram and IS terrorist groups from 2014 to 2016 to ascertain the kind of discussions about terrorist groups online. The discussions were categorized into 13 sub-topics namely; reactiveness to terrorist, fight against terrorist, government proactiveness against terrorism, advocacy for anti-terrorism, claims of attack by terrorists, threats by terrorists, recruitment by terrorists, prevalence of attacks by terrorists, condemnation of terrorist attacks, mockery of terrorism, accusation of government as ineffective, sex-slave by terrorists and victory by terrorists. Charts and tables were used to present data on discussion on the 3 above mention terrorist groups.

**Specifications table**

**Table:** 

Subject area	*Mass Communication*
More specific subject area	*Terrorism; social media; counter-terrorism*
Type of data	*Bar charts, tables*
How data was acquired	*Content analysis was conducted on online YouTube videos on ‘Al-Shabaab’, ‘Boko Haram’, and ‘IS’*
Data format	*Data is retrieved from YouTube channels and analyzed using quantitative method. (Raw, and analyzed, etc.).*
Experimental factors	*Data consists of reported incidence on Al-Shabaab, Boko Haram and IS on YouTube by News media from 2014 – 2016*
Experimental features	*Mass media channels on YouTube and other social media platforms are vital for the promotion of counter-terrorism messages and discussions.*
Data source location	*Online (YouTube) www.youtube/al-shabaab www.youtube/bokoharam*
	*www.youtube/isis*
Data accessibility	Data can be found in this article
Related research article	Tolulope Kayode-Adedeji^1,^ Olusola Oyero^2^, Aririguzoh, S^3^
	Dataset on Online mass media engagements on YouTube for terrorism related discussions.

**Value of the Data**

• The data presents helps researchers and online media communities to understand the discourse on terrorism for better future discussions on counter-terrorism.
• Educate media researchers on how best the social media can be employed in counter-terrorism while opening up a new research area of engagement.
• It helps the researchers understand how terrorist groups might be using the social media (*YouTube*), so that the government make informed decisions to regulate the online media channels [1].
• It helps inform the online mass media communities or users on the essential issues discussed about terrorist groups to determine how they can channel new discussion in scientific research towards the problem of terrorism.
• Educates the government and international security organizations on how the social media is used in promoting terrorists’ activities to enable them envisage and tackle future problems these usages could bring.

## 1 Data

The data is quantitative and retrieved from YouTube videos on Al-Shahab, Boko Haram and ISIS. Data retrieved were examined and analyzed from 2013 to 2017. Data were categorized to show government/security agents’ reactiveness to terrorists, attacks against terrorists, fight against terrorist, government proactiveness against terrorism, advocacy for anti-terrorism, claims of attack by terrorists, threats by terrorists, recruitment by terrorists, prevalence of attacks by terrorists, condemnation of terrorist attacks, mockery of terrorism, accusation of government as infective, sex-slave by terrorists and victory by terrorists [2]. The videos examined, informed the themes categorized.

**Chart 1:**
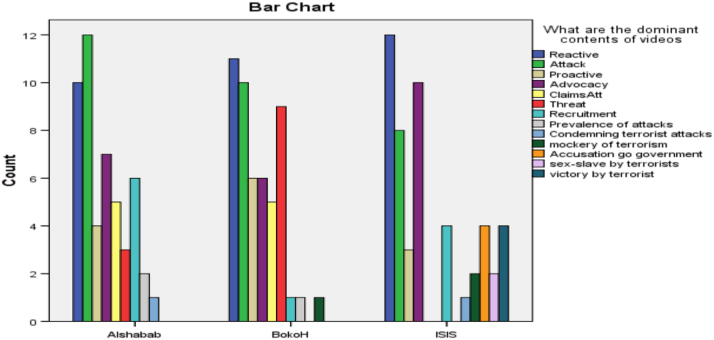
Dominant contents by terrorist organisations.

**Chart 2:**
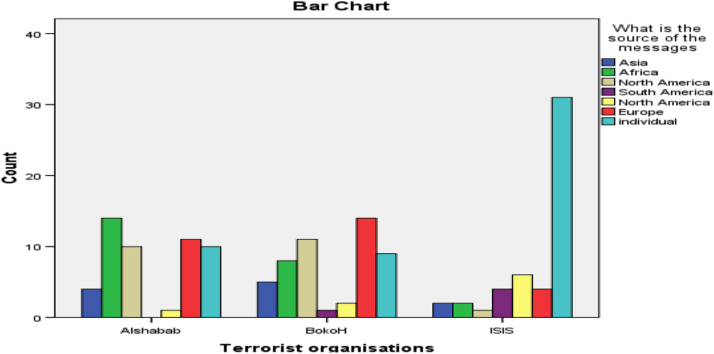
Online message source by terrorist organisations.

**Chart 3:**
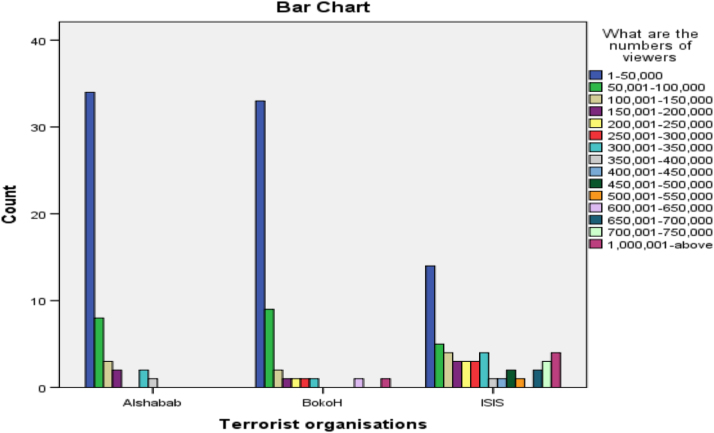
Number of online viewership by terrorist organisations.

The researcher employed the content analysis method of qualitative data by using a coding sheet to assign numbers to categories extracted from relevant videos on YouTube [3]. The coding guide specified the category and how codes were assigned numbers as specified by the coding guide. The data was analyzed using the SPSS software to generate tables and charts for visual understanding. Chart 1 presents the most dominant themes promoted by these online media outlets about the three terrorist organizations. The second chart displays the location of the online media platform against the three terrorist organizations selected, while chart third shows the numbers of viewership according to the three terrorist groups. Table 1 presents the relationship that exist between the sources of these messages and three terrorist organizations, while Table 2 examined the relationship that exist between the dominant themes in the YouTube videos and terrorist organizations using the Pearson correlation statistical tool (Chart 2, Chart 3).

**Table 1:** Correlation relationship between source of the online messages and terrorist organisations.

Correlations			
		What is the source of the messages	Terrorist organisations
What is the source of the messages	Pearson Correlation	1	0.347**
	Sig. (2-tailed)		0.000
	*n*	150	150
			
Terrorist organisations	Pearson Correlation	0.347**	1
	(2-tailed)	0.000	
	*n*	150	150

** Correlation is significant at the 0.01 level (2-tailed).

**Table 2:** Correlation relationship between dominant online content and terrorist organisations.

Correlations			
		What are the dominant contents of videos	Terrorist organisations
What are the dominant contents of videos	Correlation using Pearson	1	0.184*
	Sig. (2-tailed)		0.024
	*n*	150	150
			
Terrorist organisations	Correlation using Pearson	0.184*	1
	(2-tailed)	0.024	
	*n*	150	150

* Correlation is significant at the 0.05 level (2-tailed).

## 2 Methods

The videos examined were selected from online media channels (YouTube) using the search words of the three terrorist groups, which are Al-Shabaab, Boko Haram and ISIS. Videos were further limited to those uploaded between 2014 and 2016 by online mass media located in the seven continents. These continents include; Asia, Africa, North America, South America, Antarctica, North America, and Europe, with a maximum of 1,000,000 viewership and minimum 1000 viewership. After this, a total number of 50 video were systematically selected for each terrorist group (Al-Shabaab, Boko Haram and ISIS) to examine. For each category of terrorist group of terrorist group videos, the first video was selected after which every third video was selected until the researchers arrived at 50 for each terrorist group video. The total YouTube video then makes 150 YouTube videos. Other YouTube videos considered that met the viewership criteria, but had no specific source, were placed under the ‘individual’ source category.

## 3 Ethical considerations

The YouTube channels used granted permission by subscription to these YouTube channels to access their contents for data analysis.

## References

[1] Peter A. (2016). Cyber resilience preparedness of Africa’s top 12 emerging economies. Int. J. Crit. Infrastruct. Prot..

[2] Chiluwa I. (2016). The discourse of terror threats: assessing online written threats by Nigerian terrorist groups. Stud. Confl. Terr..

[3] Salawu A., Oyero O., Moyo M., Moyo R. (2016). A Survey of Research Foci and Paradigms in Media and Communication Master’s Dissertations and Doctoral Theses in South Africa.

